# Portal cavernoma: epidemiological, clinical, etiological and outcome profiles - a 15-year retrospective study in a University Hospital in Rabat, Morocco

**DOI:** 10.11604/pamj.2026.54.27.51543

**Published:** 2026-06-04

**Authors:** Chaima Hsain, Nawal Lagdali, Fatima Zahra Chabib, Mohamed Borahma, Fatima Zahra Ajana, Maryeme Kadiri

**Affiliations:** 1 Department of Hepatogastroenterology C, Ibn Sina Hospital, Mohammed V University, Rabat, Morocco

**Keywords:** Portal cavernoma, portal vein thrombosis, non-cirrhotic portal hypertension, thrombophilia, anticoagulation

## Abstract

Portal cavernoma is a late complication of chronic portal vein thrombosis and represents a major cause of non-tumoral portal hypertension, particularly in non-cirrhotic patients. Epidemiological data from North Africa remain scarce. This study aimed to describe the epidemiological, clinical, etiological, and outcome profiles of adult patients with portal cavernoma managed at a university hospital in Rabat, Morocco. We conducted a retrospective descriptive case series over 15 years (March 2010-May 2025), including adult patients with imaging-confirmed portal cavernoma followed at the Department of hepatogastroenterology C, Ibn Sina Hospital, Rabat, Morocco. Sixty-three patients were included, predominantly women, 73.0% (n=46). The mean age was 44 ± 13 years. Abdominal pain was the most frequent presenting symptom (46.0%; n=29). Esophageal varices were detected in 69.8% (n=44) of cases. An underlying etiology was identified in 76.2% (n=48) of patients, mainly inherited thrombophilic disorders. Long-term anticoagulation was prescribed in most patients and was associated with thrombotic stability during follow-up. Portal cavernoma cholangiopathy was the most frequent long-term complication (46.0%; n=29). Overall mortality was 9.5% (n=6). In this cohort from a university hospital in Rabat, Morocco, portal cavernoma predominantly affected young non-cirrhotic women and was mainly related to thrombotic disorders. Management relied on prolonged anticoagulation and systematic prevention of portal hypertension-related complications

## Introduction

Portal cavernoma, also known as cavernomatous transformation of the portal vein, is a late consequence of chronic portal vein thrombosis characterized by the development of a network of periportal collateral veins replacing the obstructed portal vein. It constitutes a major cause of non-tumoral portal hypertension, particularly in non-cirrhotic patients [1]. Clinical presentation is heterogeneous, ranging from incidental findings on imaging to severe complications such as variceal gastrointestinal bleeding or portal cavernoma cholangiopathy. These variables and often insidious manifestations may contribute to delayed diagnosis and management [2]. The etiological spectrum of portal cavernoma is broad and predominantly includes inherited or acquired thrombophilic disorders, autoimmune diseases, and myeloproliferative neoplasms. However, the relative contribution of these factors varies significantly across geographic regions and populations studied [3]. In North Africa, and particularly in Morocco, available data are scarce and largely fragmented, limiting a comprehensive understanding of disease patterns and outcomes. Moreover, most available studies originate from Europe and Asia, and data describing the epidemiological, clinical, and etiological characteristics of portal cavernoma in North African populations remain limited. This study aimed to describe the epidemiological, clinical, etiological, therapeutic, and outcome profiles of adult patients with portal cavernoma managed at a university hospital in Rabat, Morocco, to contribute regional data and improve disease characterization in this setting.

## Methods

**Study design and setting:** this was a retrospective, descriptive, observational study conducted at the Department of Hepatogastroenterology C, Ibn Sina Hospital, a tertiary university hospital affiliated with Mohammed V University in Rabat, Morocco. The study period extended over 15 years, from March 2010 to May 2025.

**Study population:** the source population consisted of all patients followed for portal vein thrombosis in the department during the study period. The target population included adult patients diagnosed with portal cavernoma. Among 83 identified patients, 63 adult patients with portal cavernoma confirmed by imaging were included in the final analysis. Inclusion criteria were age ≥18 years at the time of diagnosis and the presence of portal cavernoma confirmed by at least one imaging modality, including Doppler ultrasonography, computed tomography, and/or magnetic resonance imaging. Patients with acute portal vein thrombosis and those with portal vein obstruction of tumoral origin were excluded. Both patients with and without liver cirrhosis were eligible for inclusion. Given the rarity of the condition, all eligible patients identified during the study period were included, and no formal sample size estimation was performed.

**Laboratory analysis:** laboratory evaluation included routine hematological and biochemical tests performed as part of the diagnostic and etiological assessment. These included complete blood count, liver function tests, coagulation profile, and thrombophilia screening. Thrombophilia testing included protein C, protein S, and antithrombin III levels, as well as investigations for antiphospholipid syndrome. When clinically indicated, additional investigations for myeloproliferative neoplasms were performed according to institutional practice.

**Data collection:** data were retrospectively collected from patients´ medical records and computerized departmental databases. Collected variables included demographic characteristics, clinical presentation, biological parameters, radiological and endoscopic findings, etiological work-up, therapeutic management, and follow-up outcomes. Imaging modalities used for diagnosis included Doppler ultrasonography, computed tomography, and magnetic resonance imaging, while upper gastrointestinal endoscopy was performed to assess portal hypertension-related complications. Data extraction was performed using standardized data collection forms to ensure consistency.

**Definitions:** portal cavernoma was defined as the presence of multiple tortuous periportal collateral veins replacing the normal portal vein, confirmed by imaging studies. Portal hypertension-related manifestations included splenomegaly, ascites, esophageal varices, and portosystemic collateral circulation detected on imaging or endoscopy. Portal cavernoma cholangiopathy was defined as biliary abnormalities related to portal cavernoma identified on imaging. Thrombotic stability was defined as the absence of progression of portal vein thrombosis during follow-up imaging.

**Statistical analysis:** statistical analysis was performed using SPSS software. Quantitative variables were expressed as medians with ranges, while qualitative variables were reported as frequencies and percentages. Comparisons of proportions were performed using Fisher´s exact test, when appropriate, with a significance level set at p < 0.05. Results were interpreted according to standard statistical significance thresholds.

**Ethical considerations:** the study was conducted in accordance with the principles of the Declaration of Helsinki. Patient data were anonymized, and the need for formal ethical approval was waived.

## Results

**Baseline demographic characteristics:** a total of 63 adult patients with imaging-confirmed portal cavernoma were included in the analysis. The study population was predominantly female, with 46 women (73.0%) and 17 men (27.0%). The median age at diagnosis was 44 years, with ages ranging from 19 to 72 years. Baseline demographic and clinical characteristics are summarized in Table 1.

**Table 1:** baseline epidemiological and clinical characteristics of adult patients diagnosed with portal cavernoma at the Department of Hepatogastroenterology C, Ibn Sina Hospital, Rabat, Morocco, between March 2010 and May 2025 (N=63)

Variable	Value
Age; years	44 (19-72)
Female	46 (73.0%)
**Clinical presentation at diagnosis**	
Abdominal pain	29 (46.0%)
Gastrointestinal bleeding	11 (17.5%)
Ascites	5 (7.9%)
Incidental diagnosis	13 (20.6%)
**Portal hypertension features**	
Esophageal varices	44 (69.8%)
Splenomegaly	24 (37.6%)
Portosystemic collaterals (imaging)	18 (28.6%)

**Clinical presentation at diagnosis:** clinical presentation at diagnosis was heterogeneous. Abdominal pain was the most frequent presenting symptom, reported in 46.0% (n=29) of patients. An inaugural episode of gastrointestinal bleeding was observed in 17.5% (n=11) of cases, while ascites was the initial manifestation in 7.9% (n=5). In 20.6% (n=13) of patients, portal cavernoma was diagnosed incidentally during imaging performed for unrelated indications.

**Portal hypertension-related manifestations:** clinical and radiological features of portal hypertension were commonly observed. Splenomegaly was detected in 37.6% (n=24) of patients, and radiological evidence of portosystemic collateral circulation was present in 28.6% (n=18). Ascites, either clinically or radiologically documented, was observed in 15.9% (n=10) of cases. All patients underwent upper gastrointestinal endoscopy as part of the diagnostic work-up and follow-up, and esophageal varices were identified in 69.8% (n=44) of patients, reflecting the high prevalence of clinically significant portal hypertension in this population.

**Etiological findings:** an underlying etiology was identified in 76.2% (n=48) of patients. Inherited thrombophilic disorders constituted the most frequent etiological group, identified in 41.3% (n=26) of patients, mainly including deficiencies in protein C, protein S, and/or antithrombin III, either isolated or combined. Acquired etiologies were also observed, including antiphospholipid syndrome in 12.7% (n=8) of patients and myeloproliferative neoplasms in 11.1% (n=7). Porto-sinusoidal vascular disease was diagnosed in 9.5% (n=6) of cases. Associated autoimmune diseases were also reported, particularly celiac disease in 7.9% (n=5) and pernicious anemia in 3.2% (n=2). Despite an extensive etiological work-up, no underlying cause could be identified in 23.8% (n=15) of patients. The distribution of etiological factors is illustrated in Figure 1.

**Figure 1:**
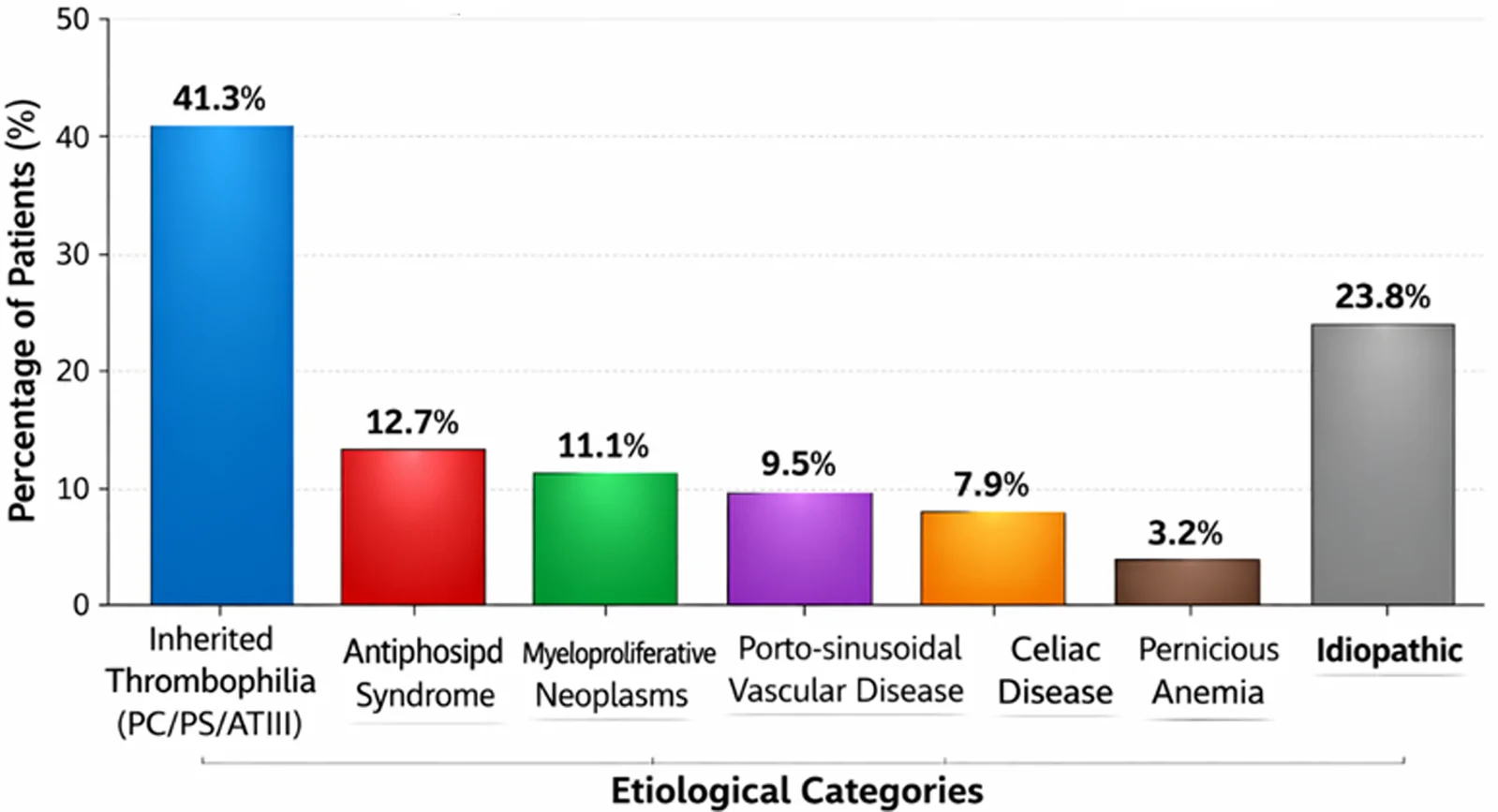
etiological distribution of portal cavernoma among adult patients managed at the Department of Hepatogastroenterology C, Ibn Sina Hospital, Rabat, Morocco, between March 2010 and May 2025 (N=63)

**Therapeutic management:** long-term anticoagulation therapy was initiated in the majority of patients, 93.7% (n=59). Among treated patients, direct oral anticoagulants (DOACs) were prescribed in 66.1% (n=39), whereas vitamin K antagonists (VKAs) were used in 33.9% (n=20). In addition to anticoagulation, all patients received primary or secondary prophylaxis of portal hypertension-related bleeding with non-selective beta-blockers 100% (n=63). Endoscopic management was performed when indicated. Surgical intervention was required in 1.6% (n=1) of patients and consisted of a splenorenal shunt.

**Thrombotic evolution under anticoagulation:** under anticoagulation therapy, the overall thrombotic course was favorable. Among treated patients, thrombotic stability was observed in 89.8% (n=53/59), whereas thrombotic progression occurred in 10.2% (n=6/59). Thrombotic progression was exclusively observed in patients receiving DOACs 15.4% (n=6/39), while no thrombotic progression was recorded among patients treated with VKAs. Although this difference suggested a trend, it did not reach statistical significance (p=0.084).

**Long-term outcomes and complications:** during follow-up, portal cavernoma cholangiopathy emerged as the most frequent long-term complication, occurring in 46.0% (n=29) of patients. Overall, 74.6% (n=47) of patients remained free of major portal hypertension-related decompensation events during follow-up. However, 41.3% (n=26) experienced at least one episode of clinical decompensation, mainly hemorrhagic complications 22.2% (n=14) and ascitic decompensation 15.9% (n=10). Because some patients experienced more than one complication during follow-up, reported percentages are not mutually exclusive. Overall mortality during follow-up was 9.5% (n=6), with deaths primarily attributed to severe gastrointestinal bleeding secondary to portal hypertension. Long-term outcomes are summarized in Table 2.

**Table 2:** long-term outcomes and complications observed during follow-up of adult patients with portal cavernoma managed at the Department of Hepatogastroenterology C, Ibn Sina Hospital, Rabat, Morocco, from March 2010 to May 2025 (N=63)

Variable	n (%)
Portal cavernoma cholangiopathy	29 (46.0%)
Hemorrhagic decompensation	14 (22.2%)
Ascitic decompensation	10 (15.9%)
Free of major decompensation events	47 (74.6%)
Overall mortality	6 (9.5%)

## Discussion

This study aimed to describe the epidemiological, clinical, etiological, therapeutic, and outcome profiles of adult patients with portal cavernoma managed at a university hospital in Rabat, Morocco. The main findings showed that portal cavernoma predominantly affected young patients with a marked female predominance (73.0%), was mainly associated with thrombophilic disorders, and was characterized by a high prevalence of portal hypertension-related complications, particularly esophageal varices. This study confirms that portal cavernoma predominantly affects young patients, with a marked female predominance (73.0%), consistent with international series on chronic portal vein thrombosis and non-tumoral extrahepatic portal vein obstruction [1,4]. This female predominance may be partly explained by the higher prevalence of inherited thrombophilic disorders and autoimmune diseases among women.

From a clinical perspective, symptomatology was mainly dominated by abdominal pain and complications related to portal hypertension, particularly the high prevalence of esophageal varices observed in 69.8% of patients, in line with previously reported data [1,2]. These findings support the systematic use of upper gastrointestinal endoscopy at diagnosis, as recommended by current guidelines [3]. These results highlight the importance of early screening and systematic endoscopic evaluation in this population to prevent hemorrhagic complications. Liver elastography showed absent to mild or moderate fibrosis (F0-F2) in the majority of evaluated cases, confirming the predominance of non-cirrhotic forms, in agreement with Western and Asian series [1,4].

An underlying etiology was identified in 76.2% of patients, mainly inherited thrombophilic disorders, followed by acquired causes such as antiphospholipid syndrome and myeloproliferative neoplasms. These results underscore the importance of a comprehensive etiological work-up in patients with portal cavernoma, as recommended by European guidelines [3]. The proportion of idiopathic cases remained comparable to that reported in the literature [1,4]. These findings support the need for systematic and standardized etiological investigations in clinical practice. Portal cavernoma cholangiopathy was the most frequent long-term complication, occurring in 46.0% of patients, a rate similar to that reported in series using systematic biliary imaging [2]. Although often asymptomatic, this complication warrants long-term clinical and radiological surveillance. This highlights the need for regular follow-up and early detection strategies to prevent biliary complications.

From a therapeutic standpoint, management was primarily based on long-term anticoagulation. The increasing use of direct oral anticoagulants reflects evolving clinical practice. Available data suggest comparable efficacy to vitamin K antagonists, with acceptable safety profiles; however, the level of evidence remains moderate, and clear recommendations are still lacking [4,5]. In our cohort, no statistically significant difference was observed between DOACs and VKAs regarding thrombotic outcomes. These findings suggest that DOACs may represent a therapeutic alternative, although further prospective studies are needed to confirm their efficacy and safety.

Overall mortality (9.5%) was comparable to that reported in adult cohorts and was mainly related to hemorrhagic complications of portal hypertension [1,4]. This underscores the importance of optimal management of portal hypertension to improve patient outcomes. Importantly, this study provides one of the few comprehensive descriptions of portal cavernoma in North Africa, highlighting regional epidemiological and etiological patterns. This study has several limitations, including its retrospective and single-center design, as well as the limited number of events for certain subgroup analyses, which may limit the generalizability of the findings. However, strengths include the long study period, systematic imaging confirmation, and detailed etiological assessment.

## Conclusion

In this cohort from a university hospital in Rabat, Morocco, portal cavernoma predominantly affected young women, most often in the absence of cirrhosis, and was mainly related to thrombotic etiologies. Clinical presentation was largely driven by complications of portal hypertension, while portal cavernoma cholangiopathy represented the most frequent long-term complication. Management was mainly based on prolonged anticoagulation and prevention of hemorrhagic complications. These findings highlight the clinical profile and outcomes of portal cavernoma in this setting.

***What is known about this topic***

- *Portal cavernoma is a late consequence of chronic portal vein thrombosis and a major cause of non-tumoral portal hypertension;*
- *Thrombophilic disorders are the leading etiological factors in non-cirrhotic patients;*
- *Data from North Africa regarding epidemiology and outcomes remain scarce*.

***What this study adds***

- *This study describes a large cohort of patients with portal cavernoma managed in a university hospital in Rabat, Morocco;*
- *It highlights a predominance of young women and a high frequency of inherited thrombophilic disorders;*
- *It shows favorable thrombotic stability under long-term anticoagulation and identifies portal cavernoma cholangiopathy as the most frequent complication*.
